# The social determinants of tuberculosis treatment adherence in a remote region of Papua New Guinea

**DOI:** 10.1186/s12889-016-3935-7

**Published:** 2017-01-13

**Authors:** Tanya Diefenbach-Elstob, David Plummer, Robert Dowi, Sinba Wamagi, Bisato Gula, Keyanato Siwaeya, Daniel Pelowa, Peter Siba, Jeffrey Warner

**Affiliations:** 1 College of Public Health, Medical & Veterinary Sciences, James Cook University, Townsville, QLD 4811 Australia; 2 Australian Institute of Tropical Health and Medicine, James Cook University, Townsville, QLD 4811 Australia; 3 School of Medicine, Griffith University, Gold Coast, QLD 4222 Australia; 4 Balimo District Hospital, Balimo, Western Province Papua New Guinea; 5 District Health Services Hospital, Newtown-Balimo, Western Province Papua New Guinea; 6 Papua New Guinea Institute of Medical Research, Goroka, Eastern Highlands Province Papua New Guinea

**Keywords:** Tuberculosis, Papua New Guinea, Treatment adherence, DOTS, Remote health, Subsistence, Witchcraft, Health systems, Health education

## Abstract

**Background:**

Papua New Guinea (PNG) is a diverse and culturally-rich country with severe infrastructural and health problems. Tuberculosis (TB) is widespread, and the number of cases with drug resistance is rising. Treatment adherence is known to be important for both effective treatment and limiting the emergence of drug resistance. The aim of this study was to construct a matrix of the factors that act as facilitators or barriers to TB treatment adherence in a remote region of PNG.

**Methods:**

The study was based in the Balimo region of the Western Province. People known to have undergone TB treatment, as well as staff involved in managing people with TB, were asked to participate in an in-depth interview about their experiences. Purposive sampling was used to identify a diverse range of participants, from different geographic locations, social backgrounds, and with successful and unsuccessful treatment outcomes. The interview data was analysed based on grounded theory methodology.

**Results:**

The study identified a range of factors that influence TB treatment adherence, with these being classified as personal, systems, and sociocultural. These factors are presented along with suggested recommendations for adaptations to DOTS-based treatment in this region. Barriers included the challenges associated with travel to treatment sites, and the difficulties of undertaking treatment alongside the daily need to maintain subsistence food production. However, facilitators were also identified, including the positive influence of religious beliefs, and high confidence in the ability of DOTS-based treatment to cure TB.

**Conclusions:**

Documenting the wide range of factors that influence treatment adherence in a severely affected remote population will assist in improving TB control. These results provide impetus for further community-based efforts aimed at improving access to TB diagnosis and treatment, and maintaining successful treatment outcomes in the face of emerging drug resistance.

**Electronic supplementary material:**

The online version of this article (doi:10.1186/s12889-016-3935-7) contains supplementary material, which is available to authorized users.

## Background

The treatment of tuberculosis (TB) is an arduous undertaking for patients, requiring six months of daily treatment with multiple drugs, and regimens of up to two years where cases are drug-resistant. The challenges of TB treatment have led to the development of the World Health Organization (WHO)-recommended directly observed treatment, short-course (DOTS) strategy for treatment [1].

There is no doubt that the use of DOTS has enabled treatment and improved outcomes for many TB patients. For example, in Ethiopia and the Solomon Islands, DOTS has resulted in increased treatment success rates and decreased treatment default rates in TB patients [2, 3]. Despite this success, DOTS typically doesn’t fully account for the influence of sociocultural and structural factors on health and healthcare delivery, which can influence TB treatment in many ways.

The worldwide emergence of drug-resistant TB (DR-TB) makes the influence of sociocultural and structural factors on treatment adherence even more important. Factors that result in delayed diagnosis and treatment interruptions increase the likelihood of both generating and selecting genetic mutations that confer antimicrobial resistance. Furthermore, the establishment of drug-resistant strains enables primary transmission of these strains to others. Understanding facilitators and barriers to treatment is therefore essential in local TB control programs.

In Papua New Guinea (PNG), TB contributes significantly to the burden of disease. In 2014, the WHO estimate of incidence was 417 cases per 100,000 people, with multidrug resistance estimated at 2.7% in new cases, and 19% in retreatment cases [4]. However, region-specific studies in PNG have suggested higher rates of TB and drug resistance, with incidence estimates of 550–1290 cases per 100,000 people, and multidrug resistance in 5–25% of patients [5–10].

Research in PNG has described a diversity of beliefs about health, illness and treatment [11]. Furthermore, a number of sociocultural and structural barriers to TB treatment have been described. These include poor health facilities, overcrowding, isolation, certain ‘unhealthy’ behaviours, and cultural beliefs [12].

The aim of this study was to identify factors influencing TB treatment adherence in the remote Balimo region of PNG. Identifying these factors is an initial step in supporting and strengthening the local TB control program, thus enabling patients to adhere to treatment, and stemming the rise of DR-TB. Furthermore, regional factors such as geographical challenges and predominantly subsistence-based lifestyles make the results of this study of interest to TB control programs across the greater PNG area.

## Methods

### Study setting

**Fig. 1:**
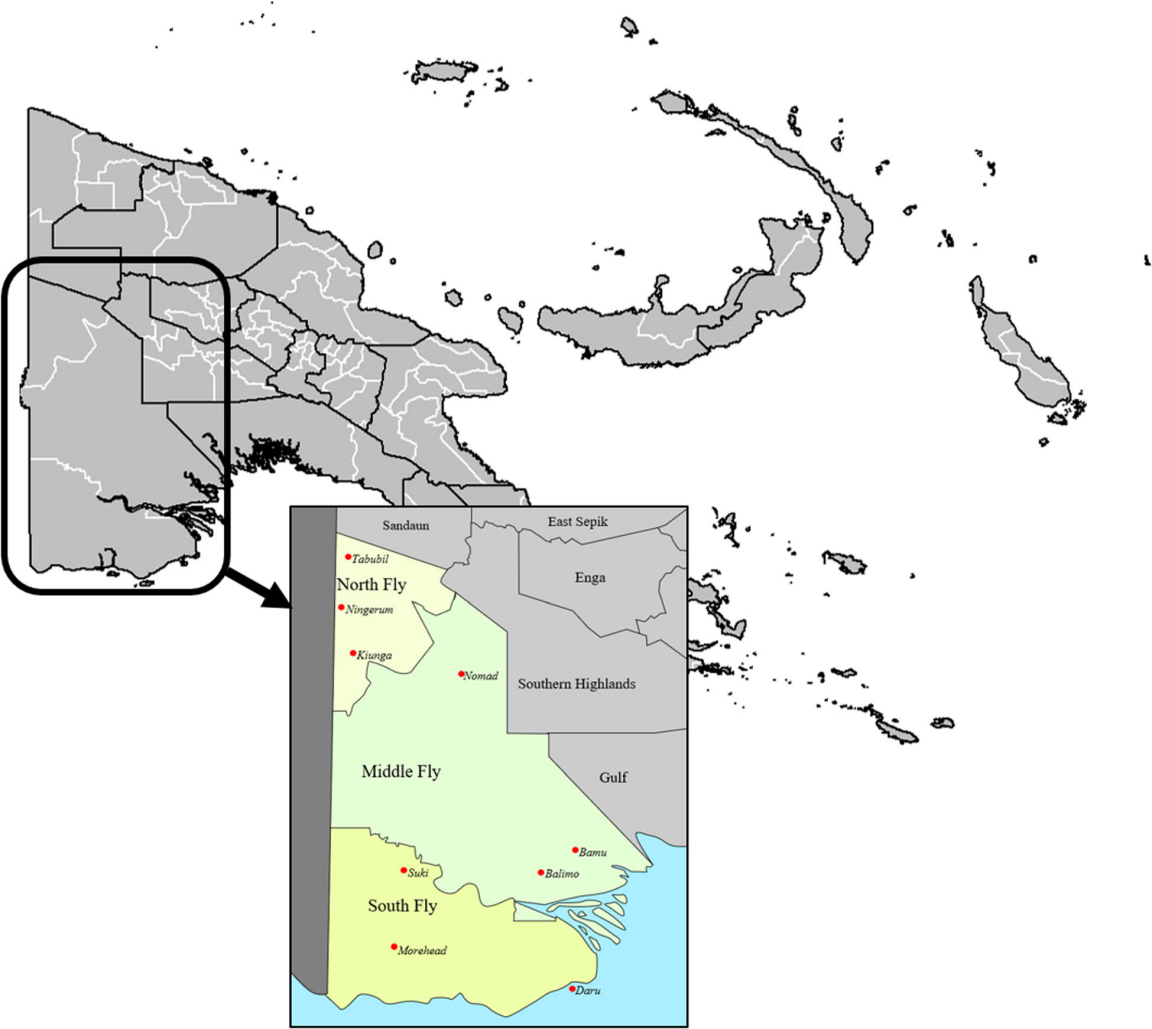
Papua New Guinea, showing Western Province, and Balimo in the Middle Fly District

The study was based in the Balimo region in the Middle Fly District of the Western Province of PNG (Fig. 1). The area has a population of about 37,000, with about 4000 in the Balimo town area. The majority of the population belong to the Gogodala local language group.

There is strong influence from colonial history in PNG, with the population predominantly identifying as Christian [13]. However, traditional beliefs and practices, including witchcraft and sorcery, are still influential in everyday life [14].

PNG has a rugged and varied landscape, with the majority of the population living in rural areas. The Balimo region is situated on the Aramia River floodplain, with Balimo adjacent to a large lagoon. Transport in the area is primarily by boat, ranging from dugout canoes paddled by hand to larger canoes, and, less commonly, in fibreglass dinghies powered by outboard motors. Road networks are extremely limited, with vehicle transport restricted to the town area. As a result, travel for purposes such as accessing health care, which may be located far from home, can be both lengthy and costly.

Balimo has two hospitals, however there is no physician. Clinical decisions are made by a health extension officer and nursing staff. Facilities such as surgery and x-ray are not available, so clinical investigations and sputum testing using Ziehl-Neelsen microscopy are used for diagnosis. Suspected TB cases must travel to Balimo, often from very remote sites, as this is the only health facility in the District able to diagnose TB and initiate DOTS-based TB treatment. Accessing TB services may therefore be particularly difficult for those who are ill, or who live in remote villages.

The majority of the population are engaged in subsistence agriculture as the primary means of food production. This involves travelling by boat or by foot to camping places and gardens, or fishing in the extensive lagoon system, as the primary means of food production. The subsistence-based lifestyle means cash income is very limited for most.

### Study design

This study used in-depth interviews and site visits to explore the lived experience of people who have undergone TB treatment. The study primarily aimed to gather patient perspectives of treatment adherence, however a small number of health workers were also interviewed to give further depth to the findings. In addition to their own experiences, interview participants acted as lay field observers, providing insights into complex social systems and structures that may influence TB control, including cultural beliefs and practices, village obligations, food security, family life, and health systems. Participants were recruited by local health workers using purposive and maximum variation sampling to identify people with a diverse range of treatment experiences. The site visits added rich observational data that enhanced the interview findings.

Interviews were semi-structured and lasted for approximately 30–60 min. Initial questions gathered demographic and social data, including age, family details, living arrangements, and education (Additional file 1). The interview body aimed to collect accounts of each participant’s individual experience with TB treatment, as well as their observations of others, in order to develop a composite model of as many factors as possible that potentially impinge on treatment adherence. The content of interviews was highly variable by design; interviews aimed to include both clinical and contextual issues, including treatment experiences; delays in diagnosis and treatment; access pathways to treatment; services and support; beliefs about the causes of TB; drug side effects; treatment adherence; attitudes and beliefs about treatment; social systems and organisation; support networks; and cultural contexts. Staff interviews focused on past and present approaches to TB treatment in Balimo, and only data relevant to treatment adherence was included in these results.

### Study population

A total of 21 individual interviews were conducted. Participants were briefed on the nature of the study, and verbal informed consent was obtained prior to each interview. Eleven interviews (including four with staff members) were conducted at Balimo District Hospital, with the remaining ten undertaken during site visits in outlying villages. Participants were given the option of being interviewed in English or the local Gogodala language. Two local community nurses acted as translators and cultural advisers in all patient interviews where required. All health worker interviews were conducted in English.

Participants interviewed about their treatment experiences ranged in age from 23 to 61, and included 11 males and 6 females. Demographic factors of the 17 participants interviewed about their treatment experiences are shown in Table 1. Where housing density was able to be identified, this ranged from 1.25 to 11 people per room. The health worker participants were aged from 28 to 51, with one female and three males, and with a broad range of education and expertise.

**Table 1:** Demographic and social factors of participants interviewed about their TB treatment experiences

Location	Urban (Balimo)	7/17 (41.2%)
	Outlying village	10/17 (58.8%)
Sex	Male	11/17 (64.7%)
	Female	6/17 (35.3%)
Age	18–30	4/17 (23.5%)
	31–40	6/17 (35.3%)
	41–50	3/17 (17.6%)
	51–60	2/17 (11.8%)
	61–70	1/17 (5.9%)
	Unknown	1/17 (5.9%)
Education	Primary (Grades 1–6)	8/17 (47.1%)
	High school (Grades 7–12)	3/17 (17.6%)
	College/Tertiary	4/17 (23.5%)
	Unknown	2/17 (11.8%)
Living status	Employed	4/17 (23.5%)
	Predominantly subsistence	10/17 (58.8%)
	Student	1/17 (5.9%)
	Home duties	1/17 (5.9%)
	Unknown	1/17 (5.9%)
Relationship status	Married	13/17 (76.5%)
	Separated	1/17 (5.9%)
	Unmarried	3/17 (17.6%)

Although the 21 interviews traced individual patient and health worker journeys, participant observations of social and cultural influences resulted in data relevant to the broader social and community context. Thus the sample for this study incorporates the broader social structures and systems, beyond the individual key informants themselves.

### Data analysis

All interviews were digitally recorded in duplicate. Interview transcripts were manually coded using open and axial coding [15]. Analytic memos documented details of emerging codes and categories throughout the coding process. Analysis was based on adapted grounded theory methodology, aiming to develop a matrix of factors and subsequently an explanatory model for the factors that influence treatment adherence [16, 17].

### Human ethics approval

The study was approved by the James Cook University Human Research Ethics Committee under approval number H5661. The Middle Fly District Health Service and the Evangelical Church of Papua New Guinea Health Service gave both verbal and written permission and support for the project. The PNG Medical Research Advisory Committee approved the study.

## Results and discussion

Standard management of TB in the Balimo region is based on the DOTS strategy. Ideally, this means that patients who are deemed infectious (i.e. sputum smear-positive for Acid Fast Bacilli), as well as those who are particularly unwell, are admitted to the hospital TB ward for two weeks, or until considered non-infectious. However, limited hospital beds means this does not always occur. The approach to treatment is therefore pragmatic and flexible out of necessity. Patients may stay in or around the Balimo township during treatment, returning to the hospital each day for treatment observation. Others return home, with their treatment observed by a treatment supporter or family member. Some patients may take their treatment unobserved, returning to Balimo only to replenish their medication.

In this study, 12/17 (70.6%) participants interviewed about their experiences with TB treatment reported never having missed a treatment dose. The interview data revealed a number of sociocultural and structural factors that may act as barriers and facilitators in TB treatment adherence. These factors were apparent based on a range of experiences, including patients who missed, who came close to missing, or were tempted to miss doses, as well as lay experience based on observation of others undergoing treatment. Influencing factors are considered from personal, systems, and sociocultural perspectives.

### Personal factors

#### Personal experience

People undergoing TB treatment in the Balimo region have a knowledge base from which they approach treatment. This is composed of personal experience, observations, interactions with others, and the development of belief systems. These influences can occur long before a person is actually diagnosed with TB, and only later become relevant to the treatment experience. For example:

Once he was commenced on treatment, he had confident that he was going to get better. But then, he is not gaining weight … He has seen other people who were on TB treatment, they gained weight, they are healthy, but not him. (42 year-old male, urban, employed; translation)

Knowledge, or the lack of it, as well as a variety of beliefs about the cause of TB, were described as influences in the non-acceptance of a TB diagnosis, and resultant difficulties in adherence:

There are times other patients who will be openly accepting that they have TB. Others will still consider the other issues like it’s witchcraft or it’s a sin. They could be that. They struggle through. (39 year-old female, urban, employed)

Such examples demonstrate the capacity of observation, experience, knowledge, and beliefs to act as both facilitators and barriers in treatment. Furthermore, differing beliefs about TB causation seem to reflect efforts to explain TB based on personal experiences, despite limited knowledge of the biomedical basis of disease. These factors may be influential throughout the treatment journey, in treatment-seeking, acceptance of a diagnosis, and adherence.

#### Responses to treatment

Physical responses to treatment influenced adherence. People undergoing treatment and health workers reported relatively few side effects, or that effects disappeared rapidly after initiating treatment. It was noted that having foreknowledge of potential side effects, such as a change in urine colour – a standard feature of treatment with rifampicin – was important. However, numerous participants reported a simple desire to get better and get on with life, with this being sufficient to overcome challenges. For example:

She developed the side effects and then husband told her, maybe this one of the cause that other people don’t like TB [treatment] … because of the side effects. And they go off the treatment. And then what she told her husband is, that’s them, but this is my life. We all have one life here so I need to complete the treatment. (41 year-old female, village, subsistence; translation)

Feeling better following treatment initiation with resultant abandonment of treatment is commonly reported in the literature [18–20]. Although this was described by two participants in this study, it appeared to be unusual. Rather, it was far more common for participants to report that feeling better encouraged them to continue treatment, even if side effects occurred.

Although responses to side effects varied, it was clear that education about potential effects was important. Furthermore, ensuring that patients understand the need to continue with treatment, regardless of whether they feel better, worse, or no change at all, should be emphasised. This is particularly important in a setting where low levels of education are not unusual (Table 1), and in combination with beliefs in sorcery and inadequate science-based knowledge of disease, may combine to undermine antibiotic treatment efforts. However, patients should also be educated that feeling worse, or developing side effects, should prompt review by a health worker.

#### Competing priorities

Competing priorities were a prominent influence on treatment adherence. Missing treatment due to work-related responsibilities was mentioned by two participants. However, for a primarily subsistence-based population such as the Gogodala people, possibly the most important consideration is food production, as described here:

… last year I left the medicine, treatment. I was busy planting bananas … That’s another reason, last year I left the medicine, I was in the bush. (39-year-old male, village, subsistence)

TB and HIV treatment adherence literature describe another consequence of food shortages on adherence, with patients unwilling to take treatment on an empty stomach due to possible side effects, increased hunger due to recovery, and believing that medications taken without food are harmful [21–23]. Concern about taking medicine without food was reported in Balimo. This was particularly when there were food shortages. Such shortages are also linked with the economic burden created by the need to be away from home for treatment:

They ran shortage of food, so they have to come [back to the home village]. It was quite expensive for them to stay [in Balimo]. (41-year-old female, village, subsistence; translation)

Where lifestyles are largely subsistence-based, food-related factors impinge heavily on health and healthcare practices. Limited access to money, and thus limited ability to purchase food instead of producing it, results in difficult choices for patients: do they continue with TB treatment with inadequate nutritional intake, a choice potentially worsened by a belief in always needing to eat with medication; or do they miss some treatment so that they can produce adequate food, with the aim of continuing treatment later? Such dilemmas will be further aggravated by the degree of understanding that a patient has about the need for treatment adherence – poor understanding may result in poor adherence due to a belief that it won’t matter, while good understanding will further increase stress for patients struggling to access adequate food.

### Systems factors

#### Health education

The health system plays a significant role in treatment adherence, through facilities, staff, and education. In Balimo, participants described the effect of education received from health workers concerning treatment adherence:

I was, you know, thinking like that [of not taking the medicines], but … these other [health] officers told me that if you miss one, one of the drugs, then you’ll have a problem … So I was honest and I completed the whole of the medicines. (36-year-old male, urban)

Although some participants said they were not told anything about TB when they started on treatment, others stated that they had been given some information about what to expect. Education is essential for understanding the importance of consistent dosing and treatment. The two participants who missed a dose due to work commitments highlight the importance of flexibility when patients can’t attend for treatment, to ensure that doses are not missed in situations when supervision is not possible. Arrangements to cover such situations should be raised with patients from the outset.

#### Health infrastructure

Accessing treatment was more difficult for people in the villages, who primarily receive care at aid posts staffed by health workers with minimal training. A number of participants described receiving a routine treatment regimen of amoxicillin and aspirin in response to a variety of complaints, without onward referral to Balimo for further investigation. There was evidence of disillusionment with such ineffective routine treatment:

And then I was given that same treatment and then I got tired of getting same medicine. There wasn’t any change. (53-year-old male, village, subsistence)

Disappointment with treatment could lead to delays in diagnosis and initiating correct treatment, with attendant risks for ongoing transmission. However, patients expressed confidence in the effectiveness of the TB drugs once they started:

He is telling the difference between the previous those who were on TB treatment [loose medications], they knew that they would not get better. They would die. But this current one [the DOTS packs], they all know that once the patient is on treatment, he’s getting better. (61 year-old male, village, subsistence; translation)

The commitment to TB treatment in the region is encouraging. However, further research would help to determine whether unsuccessful routine use of basic antibiotics and analgesics affects adherence once patients begin TB treatment.

#### Drug supply

The drug supply chain was particularly problematic in Balimo prior to the introduction of DOTS. A complex regimen of individual drugs and an unstable supply chain resulted in patients being given partial courses of medication on a first-come, first-served basis, and thus experiencing treatment interruptions when medications were unavailable. These interruptions created further problems for patients who had undergone some treatment, followed by resolution of symptoms, who subsequently couldn’t see the need to resume treatment. This comment is from a participant whose TB appears to have recurred:

I said ah, I still long time … without medicine. I cannot go back. I thought I was already better. (39-year-old male, village, subsistence)

Drug supply interruptions could become a disincentive to seek ongoing treatment, with repeated unsuccessful trips to Balimo resulting in diminished desire to continue trying to receive treatment.

Because two boxes containing an entire 6-month treatment course are now supplied for people diagnosed with TB, treatment with the DOTS packs is largely free of the supply interruptions that occurred previously. However, there was evidence of potential problems. One participant had a child who experienced a week-long treatment interruption due to a shortage of streptomycin. Furthermore, as the Balimo airport is currently closed, the need to use sea- and river-based transport has the potential to make supply less straightforward.

#### General infrastructure

General infrastructure challenges have an important impact on healthcare in the Balimo region, particularly in relation to transport. Travel in the area is primarily water-based. During dry season, when water levels are low, village access by boat is more difficult, and may entail long distances by foot over rough and swampy ground. For some, access to treatment is challenging due to other factors, including the expense of travel:

They buy fuel and they come, but most of the people they don’t have engines and motors, so they travel one or two days to paddle and come. (39-year-old female, urban, employed)

Other studies have found long travel times to be associated with poor treatment adherence [24, 25]. This is a potential problem for people undergoing treatment in the Balimo region. The challenges associated with long and difficult travel are further complicated by reliance on subsistence agriculture. The daily need for food production limits the amount of spare time that people have available for treatment-seeking. Furthermore, time away from food production can exacerbate economic burden.

### Social and cultural factors

#### Social factors: support and discrimination

Support systems were important as both facilitators and barriers to treatment adherence. Good support systems facilitated treatment:

He’s a sports man, so the people, the community, they also showed their love, and their contribution towards him. Advising him, taking care of him. They didn’t want him to, they didn’t want to lose him, to die of this disease. So they gave their moral support. (38-year-old male, urban, employed; translation)

In a setting such as Balimo, where patients are often required to spend at least some of their treatment away from home, the need to rely on others was recognised as making treatment difficult:

There was some friends, my friends, but they don’t, they didn’t look after me properly … They have their friends, families too. They look after their [own]. …. Yeah, it was difficult for them to look after me properly. (41-year-old female, village, subsistence)

Some interviewees reported family members selling goods at markets to support their treatment. Usually this was for purchasing supplemental food, but also natural remedies and hygiene products. There were also reports of physical help from family members, such as fetching household water and doing other tasks for those undertaking treatment. Others received assistance in the form of cash or foodstuffs.

Stigma-related difficulties in TB treatment have been reported in the literature [21, 26]. People may experience stigma and discrimination, and support for TB patients can be actively withheld or withdrawn. Yet, there was surprisingly little evidence in Balimo of discrimination against those with TB. In one interview it was noted that poor family support makes the treatment experience difficult:

The other time when he had shortage of those drugs. And ah, he would want to send his family members, children and wife. And when they didn’t wanted to go he would say, oh you not want me to live, so I will not go. You don’t care for me, so what the use of me going and getting medicine. (39-year-old male, village, subsistence; translation)

Additionally, another participant described discrimination from the family with which she was staying:

They are not really friendly with her. Actually, they restrict her from cooking. They don’t let her do the cooking. … They don’t want the food that she cooks, that they will eat. … They have negative thoughts toward her. (29-year-old female, urban, home duties; translation)

These were the only instances of active discrimination described in the interview data, and reasons for the low level of discrimination are unclear. Despite this, for the second participant, poor support resulted in an increased desire to complete treatment, as the medicine would ‘…bring her back to life’. One health worker speculated that discrimination may be minimised due to the cultural *wantok* system in PNG that emphasises aiding friends and family in need:

It [discrimination] happens. But I haven’t seen it as a problem here, because the cultural *wantok* system is still, it’s still there. But, but it’s changing. (38-year-old male health worker)

However, this health worker went on to say that the support provided by the *wantok* system is being challenged by a scarcity of food and income. Awareness and education were also seen as contributing to a reduction in discrimination because people had a better understanding of the underlying causes and transmission of TB.

There was limited mention of the role of treatment supporters in the interviews focused on treatment experiences, but their contribution was explored more fully in the provider interviews. Treatment supporters are an important facet of the Western Province Tuberculosis Program [27]. Family members are encouraged to act as treatment supporters and observers. Additionally, a number of villagers have been trained as treatment supporters. Their roles include giving and observing treatment, collecting medications for patients, raising TB awareness at events, and reporting to health workers in Balimo. Their role warrants further research, as in a setting where access to health centres can be difficult, the use of community-based treatment supporters is likely to facilitate treatment adherence.

#### Cultural factors: religion, sorcery, and traditional remedies

Treatment adherence in Balimo is influenced by religion and traditional beliefs. Many Gogodala people are Christian, but traditional animistic practices were important historically, and, for many, they continue to influence daily life [28, 29]. The influence of religious and traditional beliefs manifested in various ways, with factors facilitating adherence centring on religion. Simply having religious beliefs and faith in God encouraged treatment adherence:

What he is saying, that while being a [Bible] student he got this disease. Firstly he thought he was going to die. But then as he was going through Bible studies, and going through God’s word, he knew that he wouldn’t die. He would get better. So that encouraged him to continue and to take his medications. (33-year-old male, village, student; translation)

Another participant described continuing adherence so that she could subsequently focus on God’s work. Religious beliefs could also be seen as an important adjunct to biomedical treatment:

I said treatment will help me. Prayer will help me. God will heal me from my sickness. But I really want to see a change within my life. So I need both treatments. In spiritually and physically. (53-year-old male, village, subsistence)

Despite the considerable importance attached to religious beliefs by a number of participants, there was limited evidence of links between churches and TB treatment. As a strong community organisation, with more extensive networks and infrastructure than the PNG government, we feel that churches should become more involved with TB treatment in Balimo. Such involvement could especially focus on treatment adherence, for example with church leaders functioning as treatment supporters. However, churches could also act as a means of communicating the TB message throughout the population, and facilitate screening and contact tracing activities in their communities.

An interesting facet of TB treatment in Balimo was the use of multiple treatment approaches by people experiencing TB symptoms. Participants described moving between different treatment options, including herbal, biomedical, religious, and sorcery, in search of effective treatment. Use of herbal or natural remedies was reported before, during, and after TB treatment. Although not identified as a factor in adherence, the use of such remedies can delay treatment-seeking. In addition, simultaneous uses of herbal and biomedical treatments raises the possibility of adverse drug interactions.

Using multiple approaches is inadvertently encouraged when routine treatment regimens, such as the ongoing use of amoxicillin and aspirin described previously, are unsuccessful:

But ah, they only gave me pain medicine. So, the pain medicine did not help … April and May I was visiting witchdoctors, and ah, prayer peoples. At that same [time] I was getting this pain medicine, but it was not helping. (32-year-old male; village, employed)

Two participants said that people’s beliefs in witchcraft were due to a lack of understanding of TB, and it is possible that the use of multiple treatment approaches is linked to confusion about the causes and presentation of TB:

What he’s saying those people [who believe in witchcraft], they don’t fully understand what TB is, the sickness itself. So when it, ah, when person is showing signs and symptoms, they think it’s witchcraft. (39-year-old male, village, subsistence; translation)

For some with limited knowledge of TB, there also seems to be a link between belief in witchcraft and the success or failure of biomedical treatment. Participants reported either new consideration of, or new proof against, witchcraft in response to their treatment experience and observations:

When he was on treatment and he wasn’t getting better he thought of being the witchcraft. (42-year-old male, urban, employed; translation)

There was one fellow, one boy. He told me that ah, we were taking TB medicine together, TB drugs together. And he told me that, witchcraft. After he was getting better and then I told him, no, I don’t think witchcraft. What I have seen. (36-year-old male, urban)

While not surprising in such a setting, such beliefs are particularly concerning in the face of emerging drug resistance, where antimicrobial regimens will be less dependable.

Although the use of multiple treatment approaches was common, there were exceptions. One participant described a non-belief in witchcraft based on his religious beliefs:

People gave me these thoughts. People were telling me about that. Somebody magicked you. That … caused TB. People were telling me this. So they were sending me to magic people to get treatment, but I said no. I was only holding Bible. I cannot believe two gods, I said. (39-year-old male, village, subsistence)

In another case, the importance of education was highlighted by one participant, who was also a health worker, who used only biomedical treatment. However, this participant advocated an approach that encouraged treatment adherence based on education and awareness, while also taking into account the knowledge, beliefs and experiences of patients:

… [my] awareness to them, is that TB is a disease itself, its cause is by a bacteria. And it’s nothing to do with witchcraft or even with sin. It’s just like a sickness, with our malaria causes. Mosquito causes the malaria. And it has got its own treatment to get better … And it’s more got to be community-based, or person-based, not us. Yeah, they need to ask a lot of questions. Give them time to view out what TB is, then upon their positions, or upon how they respond, then we can fall in to see where we can give them health awareness … Get into the shoes how they live, what they really need, then you can pass the information. (39-year-old female, urban, employed)

In this context, understanding and awareness that fits with existing cultural norms and beliefs, and which is sensitive to the level of education of the majority of the population in the area, are key factors in adherence. Health workers in the district are already working on public health education about TB awareness, particularly at large events such as the Gogodala festival [27]. This essential education may be further enhanced by understanding the underlying sociocultural attitudes and practices present in the community.

## Conclusions

**Fig. 2:**
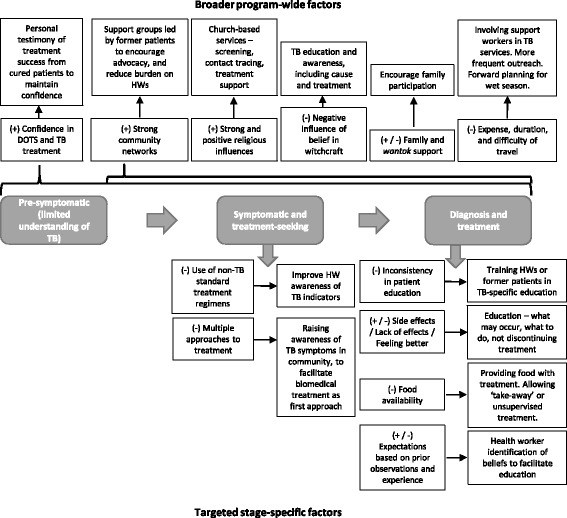
Factors influencing DOTS-based diagnosis and treatment in Balimo, and suggested adaptions that may facilitate adherence. In the diagram, factors that may act as barriers are shown with a (−) symbol, while those that may act as facilitators are shown with a (+) symbol

Overall, people in the Balimo region being treated for TB demonstrated a high level of confidence in the use of DOTS-based treatment to cure TB, when and if they were able to access such treatment. However a variety of personal, systems, and sociocultural factors had the potential to influence a person’s adherence to TB treatment. These factors are outlined in Fig. 2, with reference to stages of the treatment journey undertaken by TB patients, along with recommendations for how the DOTS approach may be adapted to address such factors in this setting. Understanding these factors is essential when adapting the DOTS control strategy to suit local conditions.

In the Balimo region, sheer necessity has already led to flexibility in the delivery of the DOTS strategy. Local adaptations to DOTS, based on the identified challenges to its implementation, should be formalised to ensure consistency in healthcare delivery. In addition, a structured approach to evaluating patient needs, based on such adaptations, would be useful in ensuring that patients are accommodated before complications arise.

Greater involvement of community networks and churches is likely to provide substantial benefit to TB services in this region. Successfully treated former patients can provide support and services to new patients, especially in a society where community links are already strong and highly valued.

Education, or the lack of it, was identified as significant in relation to many factors. Where patients receive inadequate education about their condition and treatment, this may reflect the heavy work burden faced by healthcare workers. However, educational services such as those received by new patients, could easily be provided by appropriately trained former patients.

Expanding the involvement of churches and treatment supporters may enhance treatment adherence, communication of the TB message; and screening and contact tracing activities. Furthermore, such an approach may ease the workload for hospital staff who currently have high work burdens. This should be explored through further research and focus groups, with a view to undertaking pilot projects with willing church groups and former patients.

A number of factors specific to the Balimo setting may act to facilitate TB control in this region. Confidence in the ability of the TB drugs to cure TB, especially when witnessed in others, is encouraging. This bodes well for the continued desire of people to seek care for their symptoms. In addition, for many people the successful completion of treatment encouraged them to provide testimony of their cure to others, which provides the means for establishing patient support groups in the region. Finally, the strong and positive influence of religion and churches provide a ready-made network through which TB services can be delivered.

There is no doubt that the DOTS program in Balimo is highly-regarded by both patients and health workers alike. However, the strategy is challenged in this region by a number of factors, particularly relating to patient understanding of TB, food security, transport and access difficulties, and the need for flexibility in treatment observation. In other settings, remote treatment monitoring using mobile phones has been trialled with positive results for both TB and HIV patients [30, 31]. Such an approach in this region of PNG is possible, although limited and expensive technology may be a barrier. However, this method of treatment support could be trialled with patients who do have access to mobile phones.

Community consultation will be necessary to identify the best way to implement other adaptations, such as the provision of food with treatment or other strategies to improve food security for TB patients; and ways to facilitate access to treatment when patients themselves cannot attend at Balimo.

The challenges facing the DOTS strategy in Balimo also highlight the need for broader public health strategies that emphasise reducing TB transmission, rather than relying primarily on treatment strategies. In particular, in this setting where living conditions are often crowded, and people frequently have limited understanding of the biomedical basis of disease, cough as the primary source of TB transmission is a key concern. Future work in this area should focus on this message, and investigate strategies that limit transmission when people with TB are coughing.

Emerging drug resistance is an urgent problem for the Western Province of PNG, including the Balimo region. This highlights the need for better patient education to ensure consistency in dosing, as well as increased monitoring of patients who appear unresponsive to treatment. For the community, poor treatment outcomes in drug-resistant patients may create disillusionment with treatment in others. Community education and awareness of drug resistance, including what it is and why it occurs, are essential. In addition, facilitation of treatment adherence will serve to improve treatment outcomes in the region.

## Additional file

Additional file 1: Interview guide. Semi-structured interview guide developed for the study. (DOCX 17 kb)

## Abbreviations

DOTS: Directly observed treatment, short-course
DR-TB: Drug-resistant tuberculosis
HW: Health worker
PNG: Papua New Guinea
TB: Tuberculosis
WHO: World Health Organization
