# Supplementary material for: The social determinants of tuberculosis treatment adherence in a remote region of Papua New Guinea
Source: BMC Public Health. 2017 Jan 13;17:70. doi: 10.1186/s12889-016-3935-7 (PMC5237215; doi:10.1186/s12889-016-3935-7)
Supplement: Additional file 1: — Interview guide. Semi-structured interview guide developed for the study. (DOCX 17 kb) [file 12889_2016_3935_MOESM1_ESM.docx]

Interview guide

1. Give introduction and obtain informed consent
2. Perform recording sample – state date, interviewer name, and interview number
3. Ask interviewee to state their consent
4. Obtain demographic and social data
   1. Age
   2. Sex
   3. Clan
   4. Birthplace
   5. Father’s birthplace and clan
   6. Mother’s birthplace and clan
   7. Siblings / family structure
   8. Living arrangements (present and past)
   9. Religious background
   10. Employment history
   11. Socioeconomic background
   12. School history / level of education
5. Interview body
   1. Please tell me about your experience with TB.
   2. When did you first notice illness or that something was wrong?
   3. When were you told that you had TB?
   4. Please tell me about your experience with TB treatment.
   5. What has happened to you since you completed treatment?
